# Metasurface-assisted bioelectronics: bridging photonic innovation with biomedical implants

**DOI:** 10.1038/s41377-025-02072-w

**Published:** 2025-11-24

**Authors:** Mohammad Mohammadiaria, Shashi Bhushan Srivastava

**Affiliations:** 1 Independent researcher, Pavia, Italy; 2 Henry Ford Health + Michigan State University Health Sciences, Detroit, MI USA; 3 Henry Ford Health, Department of Ophthalmology, Detroit, MI USA

**Keywords:** Optics and photonics, Optical materials and structures

## Abstract

Wireless cellular stimulation has been widely applied for bioengineering and bidirectional communication with the brain. Different technologies, such as photoelectrical stimulation as an alternative to optogenetics, have emerged for a wide range of remote therapeutic applications using light. Metasurfaces enable pixel-wise control of electric field distribution by engineering absorption and wavefront shaping, with responses tuned to incident light polarization, frequency, and phase, offering precise stimulation and wireless control in retinal, cochlear, and cardiac implants. Moreover, by leveraging terahertz (THz) band patches, reconfigurable metasurfaces controlled via FPGA and holography, and virtual reality-assisted designs, these interfaces can revolutionize bioelectronic medicine.

## Abstract (web summary)

Metasurfaces enable wireless stimulation and polarization-sensitive therapies in biomedical implants, supporting THz power transfer and photobiomodulation for applications in retinal, cochlear, cardiac, and cancer-targeted treatments.

## Graphical abstract

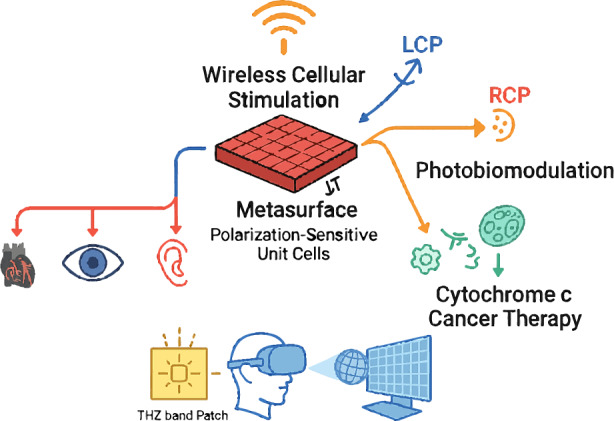

## Introduction

Bioelectronics involves the interaction of electronic devices with biological processes, including stimulation, sensing, and monitoring, making healthcare more efficient and responsive, for example, medical implants, neural interfaces, biosensors, bioelectronic medicines, and tissue engineering. Most implants rely on electrical signals with wires for activation, which results in invasive treatments along with several limitations of spatial and temporal precision. It can be mitigated by controlling the bioelectronic devices via light stimulation, sensing, and monitoring. Metasurfaces, an array of sub-wavelength nanostructures, open up possibilities to manipulate electromagnetic waves through the scattering phenomenon. Metasurfaces can be integrated into medical implants for strong light emission, manipulation, and detection for neural stimulation, color filtering for retina implant^1^, and precise detection. In this context, the terahertz (THz) band frequency regime enables both high spatial resolution and more tissue penetration due to the near Infrared (IR) wavelengths while utilizing both light intensity and polarization^2,3^. Its non-ionizing nature and compatibility with metasurface-based wavefront control make it well-suited for label-free imaging and localized stimulation^4^, though potential off-target effects from thermal diffusion and field spread warrant careful spatial control^5^. Furthermore, modulation of light polarization, when coupled with anisotropic metasurface antennas, enhances light propagation and penetration in aqueous and cellular environments, offering a promising strategy for effective cellular stimulation^6^. Such enhanced light-tissue interaction is highly advantageous in neural interfaces, particularly in brain-computer interfaces (BCI) targeting visual^7^, auditory-cortex^8^, or the neocortex, to cure a wide variety of neurological, physiological, and immunological conditions^9,10^, metasurface-based patches as nano antennas could play a crucial role in such neural implants^11,12^. For less invasive applications, such as subretinal, cochlear, and cardiac implants, a wireless system based on metasurface technology is especially valuable and can be integrated with existing platforms such as photovoltaic and piezoelectric transducers^13^. These innovations exemplify the broader evolution of implantable technologies, motivated by the clinical need for precision, minimal invasiveness, and improved energy efficiency in stimulating excitable tissues.

**Fig. 1:**
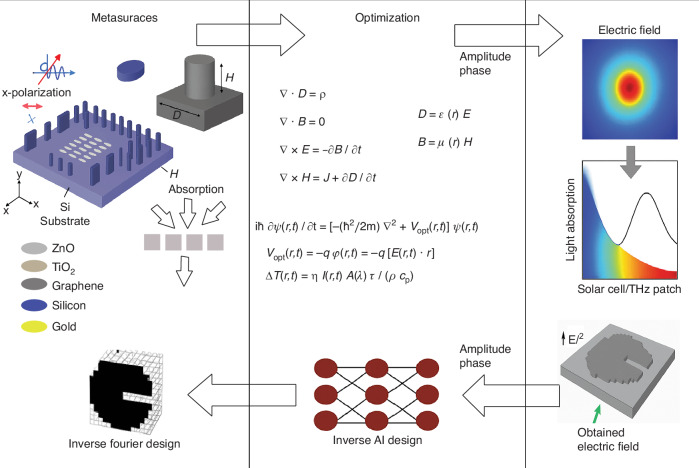
**Schematic of metasurface-assisted bioelectronic platforms spanning the visible to THz-near-IR range**. Engineered with optimized materials and geometries (e.g., Titanium oxide (TiO_₂_), Zinc oxide (ZnO), Au), these metasurfaces enhance optical absorption and electric field localization. In the visible regime, they couple with photovoltaic and organic hybrids for efficient photoelectrical stimulation, while at THz frequencies, they enable label-free imaging, photothermal release, and frequency-selective modulation. Their planar, flexible design supports wireless, minimally invasive biointerfaces

In bioelectronic platforms, we utilize metasurfaces, a versatile interface between electromagnetic fields and biological systems, to enhance optical absorption, electric field localization, and wavefront control. Depending on the application wavelength, ranging from visible to THz or near-IR, metasurface geometry, material composition (e.g., TiO_2_, ZnO, gold), and anisotropy are optimized to achieve maximal electromagnetic coupling. In the visible regime, metasurfaces integrated with photovoltaic or organic semiconductors can amplify photoelectrical stimulation for neural or cardiac interfaces. At THz frequencies, metasurfaces facilitate label-free tissue imaging, thermal modulation, and localized drug release via photothermal or phonon-driven mechanisms. Figure 1 illustrates a metasurface optimization framework for bioelectronic stimulation platforms, where both material composition (e.g., ZnO, TiO_2_, and other metal oxides) and nanostructure geometry are tuned to maximize absorption in the visible or terahertz (THz) spectral range. The substrate could also be silicon, with different combinations depending on the application to control the surface electric field and potentials. The design process involves forward Maxwell simulations to obtain the local electric field enhancement, which is then coupled to a time-dependent Schrödinger equation describing carrier dynamics and exciton generation near the metasurface-semiconductor interface. The resulting electric field profiles inform the selection of metasurface geometries that yield maximal near-field enhancement, tailored for integration with organic hybrid layers and solar cell substrates. In addition, inverse Fourier transforms enable the remapping of target electric field distributions, facilitating AI-driven inverse design approaches inspired by holographic systems, as shown in Fig. 1^14^. These optimized platforms support efficient photoelectrical stimulation of excitable cells by transducing light into *capacitive* or *faradaic* currents, offering wireless and spatially precise alternatives to traditional electrode-based implants.

The need for precise, non-invasive, and energy-efficient stimulation of excitable tissues has driven the evolution of biomedical implants. Traditional electronic interfaces, though effective, are limited by spatial resolution, long-term biocompatibility, and reliance on wired power delivery. Metasurfaces, engineered from materials such as gold, graphene, TiO_₂_, or ZnO, offer a promising alternative due to their ability to manipulate and localize electromagnetic fields across the visible to THz ranges. When tailored for integration with organic hybrid layers or solar cell substrates, these metasurface platforms enable wireless, spatially resolved stimulation by converting light into capacitive or faradaic currents. This approach supports energy-efficient, minimally invasive bioelectronic interfaces that overcome key limitations of conventional electrode-based systems. Biocompatible metasurfaces, often based on gold, graphene, or polymer composites, support safe interfacing with neural and soft tissues^15,16^. Metasurface antenna patches have already been used as power sources and for data transfer to neural implants^17–20^. In fact, direct neural modulation of the visual and auditory cortex facilitates both the treatment of blindness and hair loss issues^21^. This could be more advanced through neural implants embedded with metasurfaces. In addition, it has been shown that label-free THz imaging has enabled large-scale brain imaging^22^. Holography is one of the examples of the use of metasurfaces for virtual reality. There have already been several reviews on the applications of metasurfaces in healthcare and bioelectronics^23–25^. Recent studies have demonstrated that metasurfaces operating in the terahertz (THz) regime, particularly those incorporating 3D Dirac semimetals, can support tunable hybrid plasmonic waveguiding with strong temperature and Fermi-level-dependent modulation in the mid-infrared range^26,27^. Such tunable waveguides offer promising avenues for dynamic control in bioelectronic interfaces, enabling adaptable signal routing and localized stimulation in implantable platforms. In this perspective, we begin by reviewing the principles of metasurface design and their integration with bioelectronic implants. We then examine specific applications in neural, auditory, and cardiac systems, focusing on how metasurfaces enhance light-tissue interactions and enable wireless bidirectional cellular communication. We also explore emerging approaches involving THz-to-visible conversion, piezoelectric integration, and CMOS-based metasurface imaging. Finally, we propose future directions for THz band optogenetic-compatible implants and miniaturized artificial intelligence (AI) assisted bioelectronic metasurface implants.

**Fig. 2:**
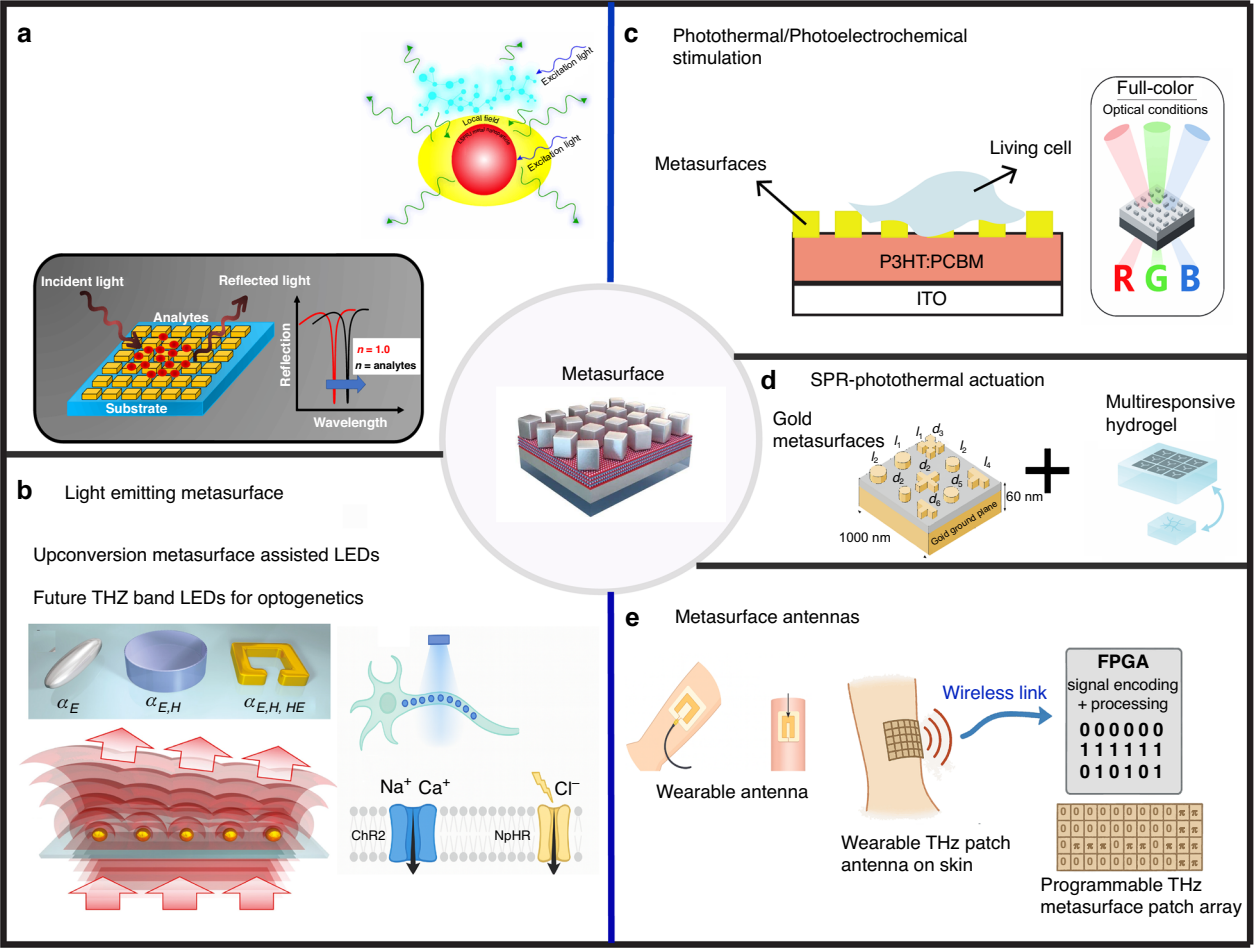
**Structure and applications of metasurface devices**. **a** Surface plasmonic resonance (SPR) sensing and enhanced fluorescence sensing; adapted with permission from ref.©^38^ Dasgupta and Ray., published by 2025 Frontiers Media S.A. **b** Metasurface-based up-conversion devices for implantable LEDs for optogenetic applications; adapted with permission from ref.©^40^ Isabelle Staude et al., published by De Gruyter, Berlin/Boston. **c** Implantable photovoltaic devices for retinal implants. **d** SPR-based photothermal actuators hybrid with multi-responsive hydrogels for tissue engineering and cell stimulation, having potential for cochlear implants. **e** Programmable implantable metasurface antennas for biomedical applications such as cardiovascular disease treatments

One of the most promising platforms for metasurface integration is photovoltaic bioelectronic systems, which enable the engineering of both light absorption and processing, as well as spatiotemporal resolution of stimulation. In this regard, it is worth briefly reviewing the photoelectric stimulation mechanism before going to the integration with the metasurface platforms. Photovoltaic substrates have been applied successfully for photoelectrical and safe stimulation of cells (such as neurons, cardiac cells, and cancerous tissues) as an alternative to optogenetics, such as subretinal implants for in vivo retinal prosthesis^21,28,29^. In this technology, photoinduced electrical charges are separated, allowing for the induction of photovoltages and currents for photo-capacitive and photo-faradaic cellular stimulations^30^. Another type of photoelectrochemical cellular modulation is based on organic polymer hybrids for modulation of intracellular reactive oxygen species (ROS) level^31,32^. Recently, embedding metasurfaces with photovoltaic substrates has been shown to enhance light absorption for boosting cellular stimulation^33^. In this application, metasurfaces are utilized for color filtering and enable the absorption of specific wavelengths^34^. Moreover, recently, color conversion from the THz band to the visible light regime has been demonstrated to be possible using nanocrystal structures and metasurfaces^35,36^. This opens new doors for THz band light sources for biomedical implants and optogenetic applications. Finally, the integration of metasurfaces as absorbers on CMOS (Complementary Metal-Oxide-Semiconductor) pixels has been demonstrated to be possible for producing THz band imaging^37^. This directly guides us in the implementation of CMOS-based retinal implants with a THz band working regime. Additionally, CMOS-based imaging devices with submillimeter working frequencies have also been designed and could be useful for biomedical applications. Figure 2 shows different applications of metasurfaces in biomedical devices, including fluorescent-enhanced SPR sensing^38^, upconversion light-emitting diode (LED) implantable devices useful for future optogenetics^39,40^, metasurface-assisted organic solar cell for photoelectrochemical cellular modulation, SPR-based photothermal stimulation with THz signal based on metasurface antennas, and metasurface antenna patches for biomedical applications. Complementing these optical systems, piezoelectric materials enable mechanical-to-electrical energy conversion for cellular stimulation and therapeutic modulation.

In addition to photovoltaic materials, piezoelectric transducers offer an alternative route for bioelectronic modulation by converting acoustic waves into electrical signals, enabling both neural stimulation and intracellular ROS regulation in cancer therapy. One example is an intraocular implant for retinal prosthesis and piezo nanomaterials for sonodynamic cancer therapy^41^. Another example of using piezoelectric-based implants is for hair cell repair, intraocular implants, and MEMS applications. The integration of metasurfaces into piezoelectric nanogenerators could be beneficial, in addition to metasurfaces integrated for bio-mems applications and cellular stimulation. Furthermore, conversion of the THz to acoustic waves is another way of ultrasound generation for the purpose of cellular stimulation^42^.

## Applications of metasurface-assisted bioelectronics

In recent times, a significant interest has developed in utilizing metasurfaces in the field of bioelectronics at various stages of healthcare. Still in its early stages of development, metasurfaces can become an essential bridge between photonics technology and biomedical implants. Here, we present several applications of metasurfaces to assist the effective use of bioelectronics. In the following sections, we highlight key emerging applications where metasurface-enabled platforms enhance functionality, starting with wireless THz-based modulation.

## I. Augmented interfaces and emerging applications

### Terahertz band patches for wireless biointerfaces

One of the technologies to convert THz band signals for cellular modulation is through thermal stimulation based on free-standing carbon nanotubes. This technology could be utilized for THz imaging and camera patches^43^. This, in fact, could be used for both the modulation of cells in different organs, such as hair cells and retinal cells, paving the way to cure blindness, hair loss, cancer, and cardiovascular disease treatments based on photothermal stimulation of cells. Furthermore, there is also another approach to convert the THz signal to mechanical changes through mechanical vibration and phonons^44^. In parallel, holographic metasurfaces integrated with virtual reality (VR) systems are being explored to enhance neural rehabilitation, particularly for restoring sensory functions such as vision and hearing.

### Holography and virtual reality integration

Holographic metasurfaces combined with VR-based neural training can enhance rehabilitation strategies for sensory prosthetics, particularly in restoring vision and hearing functions^45–47^. While conventional VR metasurfaces primarily operate in the visible to NIR (Near-Infrared) spectrum, emerging designs in the terahertz band are enabling novel functionalities such as high-resolution holography, secure biometric sensing, and ultra-fast wireless communication that may complement future VR systems. Virtual reality (VR) offers numerous applications in rehabilitation, providing interactive environments for physical, neurological, and cognitive recovery^48^. It is widely used in stroke and spinal cord injury rehabilitation^49^, helping patients regain motor function through engaging exercises. VR also aids in cognitive therapy for memory and attention deficits and provides exposure therapy for psychological conditions like Post-traumatic stress disorder (PTSD) and anxiety^50^. In the current scenario, the rehabilitation program is an essential component of the retina implant to keep track of learning modules, including the mental health of patients. Auditory Training can create immersive environments where patients practice distinguishing sounds in noisy, realistic scenarios^51^. This helps improve auditory processing, especially for people with hearing aids or cochlear implants. Spatial hearing Rehabilitation can simulate 3D sound sources to help patients localize sound better, retraining spatial hearing skills that often degrade with hearing loss. In this context, Field Programmable Gate Array (FPGA)-controlled metasurfaces offer dynamic programmability of optical responses in real-time, allowing adaptive modulation of light for bioelectronic implants, including polarization-tuned stimulation and feedback-based therapy optimization^52^. In this regard, ultrathin optical elements such as lenses embedded with metasurface phase-change materials and nanoelectromechanical actuation enable adjustable focal lengths and programmable peripheral defocus modulation to mimic eye accommodation^53^. Such adaptive devices can provide on-demand correction for myopia^54^, hyperopia, and presbyopia, while simultaneously projecting therapeutic light patterns to modulate ocular growth and slow the progression of myopia. This platform represents a potential paradigm shift toward smart, wearable vision-correction devices, offering both optical compensation and adaptive neural stimulation within a single, reconfigurable system, advancing beyond previous approaches^55^.

## II. Neuromodulation and sensory interfaces

### Metasurface-assisted photovoltaic and optoelectronic biointerfaces

Upconversion devices based on lanthanide compounds are used to convert terahertz (THz) band signals into photons that are usable for highly efficient solar cells and imaging devices. Sum-frequency generation in a nonlinear optical crystal is another method for converting THz-band and mid-telecom band IR photons to near-IR band usable for the visible absorption spectrum in solar cells or Charge-Coupled Devices (CCDs). A semiconductor-based upconversion technique is the third technology that absorbs THz signals, first absorbed with a detector, to drive a Light-Emitting Diode (LED) and emit visible-range light^56^, which could be very promising for both optogenetic technology and photovoltaic stimulation, where a solar cell has been implanted for photoelectrical stimulation. ZnO/(Antimony (Sb) and nitrogen (N)) quantum well materials have been used for THz absorption and have been successfully shown to be useful for optoelectronic applications^56,57^. Moreover, the metasurface can be integrated into hydrogel platforms either as a flexible top-layer coating or embedded within the hydrogel matrix via transfer printing and in situ polymerization. While metasurface-assisted photovoltaic platforms enable wireless stimulation with high spatial resolution, their penetration depth remains limited, restricting their use in deeper tissues such as the myocardium or inner ear. In contrast, THz-mediated approaches offer deeper tissue modulation due to reduced scattering and absorption in biological media; however, they introduce thermal safety concerns, especially for chronic implants, where repeated heating may induce tissue damage or inflammatory responses. One promising compromise involves THz-induced modulation of hydrogel permeability, enabling on-demand, frequency-selective ion release for the controlled stimulation of excitable membranes in cardiac, retinal, and cochlear tissues. Nevertheless, the stability of such hydrogel and metasurface composites, as well as the spatiotemporal control over ion flux in vivo, remains underexplored.

### Nanostructured metasurfaces in retinal implants

Retinal implant research has experienced significant advancements in recent years, transitioning to clinical trials in humans. However, the response of implants is limited to only a low visual acuity and the perception of black and white spots. Further research is required for spatio-temporal resolution and color vision. Metasurfaces could potentially manipulate the light amplitude, phase, and polarization to achieve these goals. Organic photovoltaic devices can become a game changer by enabling an integrated thin film for the creation of metasurfaces, for example, metal oxides (ZnO)^58^. By optimizing light absorption and guiding photonic energy, metasurface-assisted photovoltaic retinal implants enhance spatial resolution and improve energy efficiency, thereby overcoming limitations in current subretinal prosthetics. As briefly mentioned, the integration of metasurface structures into a photovoltaic substrate is effective in photoelectrical stimulation of cells, which could be particularly important for subretinal implants. The second application of metasurfaces is their advantages in multifunctional meta-lenses for bidirectional communication as a tool for both sensing and stimulation. Such metalenses enable color filtering and increase visual acuity. One example is reconfigurable neuromorphic-based metasurfaces^59^. There are some examples of utilizing reconfigurable metasurfaces for color filtering^57,60^, and like photosensitive materials such as perovskite nanowires for color filtering^61^. This principle of wavelength-selective control has also inspired emerging designs for neurosensory modulation. Extending this concept into the terahertz regime, recent advances explore how engineered metasurfaces can convert THz energy into mechanical or acoustic stimuli, offering new opportunities for remote, high-precision bioelectronic modulation.

### Metasurface-assisted piezoelectric biointerfaces

Conversion of the THz band signal to ultrasound through its thermal effect and phonon vibrations is an interesting growing field, which could be applied for electrical cellular stimulations as well. For this application, the first THz signal is converted into an ultrasound, and then the ultrasound signals can be employed to stimulate cells through piezoelectric materials, such as ZnO. This could be achieved through an implantable THz antenna already implanted inside the body. Finally, metasurfaces coupled to a piezoelectric nanogenerator enable us to harvest ultrasound-generated signals from a THz absorber and convert them into electrical power, which could be beneficial for piezoelectric-based biointerfaces. Building on this dual capability, metasurface-enabled systems can also support auditory modulation by delivering localized, wireless stimulation in cochlear implants.

### Optoelectronic cochlear implants

Metasurface-enabled photoelectrical stimulation or wireless patches can offer a high-fidelity alternative to traditional cochlear implants by modulating and sensing ionic currents through designed bioelectronic interfaces to optimize auditory information processing^62^. One application is increasing cochlear fidelity by adjusting potassium ions to enhance auditory information. One possible approach for such an application is through faradaic potassium ionic current modulation. In this configuration, a metasurface patch could convert a THz band signal to electrical power for driving a Palladium-based device for hydrogen peroxide ion delivery^63^. Micro-Electro-Mechanical Systems (MEMS) actuators hold significant potential for dynamic electrical stimulation of cells. Beyond mechanical positioning, MEMS platforms can integrate microelectrodes or piezoelectric elements that generate localized electric fields in response to controlled deformation^64^. This enables us to have precise, tunable stimulation regimes, offering advantages in applications ranging from neural modulation to cardiac pacing. Furthermore, coupling MEMS actuators with metasurface structures introduces the possibility of combined electrical and photothermal stimulation, expanding the toolbox for advanced, adaptive bioelectronic interfaces. This convergence of modalities paves the way for targeted neural and cardiac applications, where metasurfaces engineered with nanophotonic materials enhance the efficiency of photovoltaic stimulation in excitable tissues.

### Modulation of neural and cardiac activity

Metasurfaces engineered with nanophotonic materials enhance the efficiency of photovoltaic stimulation in excitable tissues. Hybrid approaches incorporating photothermal and photovoltaic effects enable precise modulation of neuronal and cardiac excitability. There are several cardiac diseases, such as arrhythmia and heart failure, that could be treated through cardiac tissue stimulation^65^. One example of such an application is based on metasurface-assisted implantable bioreactors, which could be integrated into the cardiac tissues^66^. In this regard, THz band patches could be applied to power pacemakers and future antennas for wireless cardiac electrotherapy^67^, used to electroporate cardiac cells for arrhythmia treatments, and integrated with photovoltaic cells for tissue regeneration^68^. Metasurfaces can be engineered to transfer terahertz or infrared energy precisely to cardiac tissues or deliver drugs or ions through a hydrogel^39^, potentially enhancing the selectivity and efficacy of electroporation-based ablation^69^. Beyond stimulation and ablation, the unique spectral properties of THz radiation have also enabled its use in biosensing applications.

### THz spectroscopy for biosensing and military inspections

Several biomolecules have a THz fingerprint, such as skeleton movements and low-frequency internal motions, which allows THz spectroscopy for the precise and nondestructive detection^70^. Biosensors embedded with metasurfaces facilitate real-time, label-free monitoring of biochemicals^71,72^, monitoring of human respiration^73^, heart rate variability monitoring^74^, monitoring cellular and tissue responses^75^, offering novel opportunities for real-time monitoring of physiological body parameters such as glucose^76^, blood hemoglobin counts^77^, eye disease detection^78^, skin cancer detection^79^, ionic currents from skin such as calcium and potassium levels^80^, protein sensing^81^, and molecular imprinted polymers (MIP) based cortisol sensor^82^ for the measurements of stress responses, which could be very promising for future multiplexed metasurface-based wearable devices. Expanding beyond physiological sensing, metasurface platforms are increasingly being developed for wireless communication, power harvesting, and real-time data transfer.

### Wireless communication, power transfer, and THz-assisted IoT

Terahertz (THz) metasurfaces enable ultra-fast, low-power communication between implanted devices and external monitoring systems^83^. Their high spatial resolution also allows for localized bioelectronic stimulation without the need for invasive wiring. Moreover, the bidirectionality of modern bioelectronic platforms, combined with high-speed communication and cyber-physical systems embedded with metasurface antennas, makes the implementation of IoT-based, patient-specific health monitoring systems both feasible and promising^84^. Integrating metasurface-enabled systems into cyber-physical health networks also raises questions around data security, real-time decision-making, and regulatory pathways for personalized implants.

**Fig. 3:**
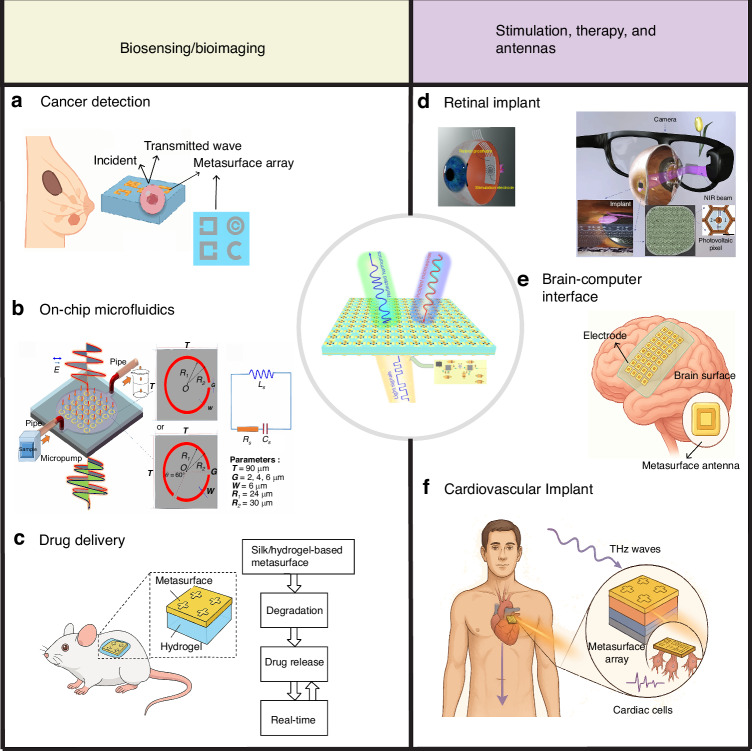
**Implantable metasurface-based devices for stimulation, biosensing, and virtual reality applications**. **a** Metasurface–integrated antenna for breast cancer detection based on implantable metasurface devices. **b** Metasurface-based microfluidic device for in vitro sensing, adapted with permission from ref. ^87^©, Geng, Z et al., published by Springer Nature Limited. **c** Implantable metasurface hydrogel for drug delivery. **d** Conceptual schematic of a waveguide-based AR display system utilizing three types of meta-devices and a subretinal prosthesis device; adapted with permission from ref. ^89^©, Ramirez, K. A. et al. published by BioMed Central Ltd unless otherwise stated. Part of Springer Nature. **e** Brain-computer interface device embedded with metasurface. **f** Metasurface-Based Bioelectronic Implants for Cardiovascular Diagnosis and Therapy

Representative examples of metasurface-assisted bioelectronic platforms are shown in Fig. 3, highlighting their versatility across diagnostic, therapeutic, and sensory applications. Figure 3 presents a range of implantable and wearable metasurface-based devices designed for stimulation, biosensing, and virtual/augmented reality applications. Panel (a) illustrates a plasmonic metamaterial-integrated antenna used for breast cancer detection, demonstrating the potential of implantable metasurface devices^85,86^. Panel (b) shows a metasurface-based microfluidic platform for in vitro biochemical sensing^87^. Panel (c) depicts an implantable metasurface-hydrogel composite designed for targeted drug delivery applications^88^. Panel (d) presents a conceptual schematic of a waveguide-based augmented reality (AR) display system integrating three types of metadevices, including a subretinal prosthesis for visual interface applications^89^. Panel (e) highlights a brain-computer interface (BCI) system capable of incorporating metasurface structures for enhanced signal transmission^11^. Finally, Panel (f) demonstrates a metasurface-based antenna interfaced with a cardiovascular implant, showcasing its potential for wireless biomedical communication^19,90^.

### Outlook and future trends

As discussed, utilizing metasurfaces as smart antennas offers a wide variety of freedom in both selective remote cellular sensing and modulation due to the tunability of their structure and their selective frequency responses. Particularly in retinal prostheses, their role in the enhancement of optical absorption (when integrated with a photovoltaic device), processing visual information, and FPGA-based programmable metalenses for advanced virtual reality-based devices to correct visual abnormalities is crucial. Additionally, coupling metasurface antennas to traditional bioelectronic interfaces for powering and driving bioelectronic actuators, as well as for ion and drug delivery, holds significant promise for both tissue engineering and cancer therapies. Furthermore, upconversion devices enable THz band implants for optogenetics and sonogenetics in the case of THz signal conversion to ultrasound signals. Moving forward, future research should focus on optimizing integrated AI-driven adaptive control and expanding multimodal stimulation capabilities that empower cardiac implantable treatments. The role of machine learning will be crucial in the rehabilitation program for patients after implantation, especially in adapting to the foreign object in the human body. Metasurfaces in an evolved and stabilized form are going to be an integral part of bioelectronic implants for the precise creation, modulation, and detection of light interacting with a human body with AI-driven feedback to achieve a biocompatible solution to healthcare.

**Fig. 4:**
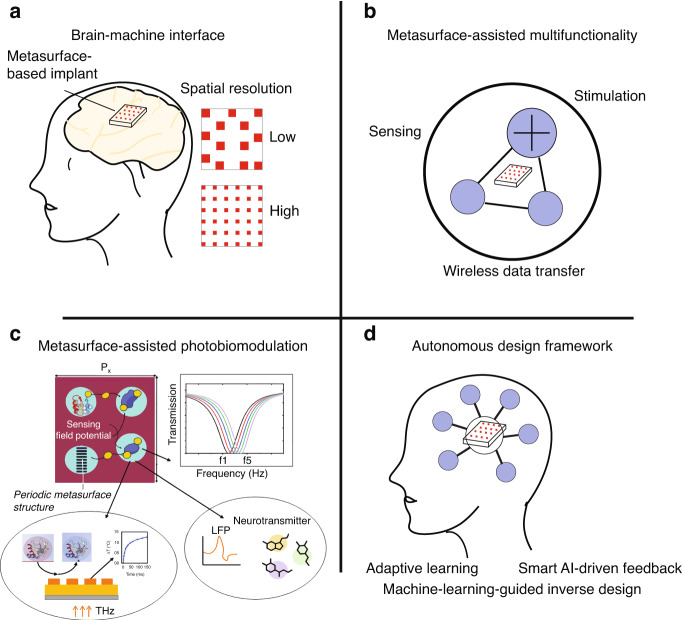
**Schematic illustration of future directions in metasurface-assisted bioelectronics**: **a** A metasurface-enabled brain implant for targeted neural stimulation; **b** Multifunctional metasurface platforms integrating sensing, stimulation, and modulation capabilities; **c** Metasurface-assisted photobiomodulation for precise, non-invasive tissue interaction across visible to THz bands for both neuromodulation and cancer therapy; **d** An autonomous AI-driven design framework optimizing metasurface geometry and material properties for specific neuromodulation application

Building upon recent advances in metasurface-assisted bioelectronics, emerging directions are set to reshape the landscape of neural modulation, cancer therapy, and implantable interfaces. For instance, upconversion-based devices are paving the way for terahertz (THz) band implants that can transduce THz radiation into ultrasound signals, enabling novel optogenetic and sonogenetic interventions. Future research should prioritize the development of integrated, AI-driven adaptive control systems to enhance multimodal stimulation capabilities, particularly in the context of cardiac implants and personalized therapeutic strategies. Furthermore, machine learning will play a pivotal role in rehabilitation programs, assisting patients in adapting to implanted devices and improving long-term biocompatibility. As metasurfaces evolve toward greater stability and functional precision, they will become essential components of bioelectronic systems, enabling the controlled generation, modulation, and detection of light within the human body, all under AI-mediated dynamic feedback loops to ensure safe and effective treatment^91^. The integration of metasurfaces with closed-loop systems and artificial intelligence enables real-time adaptability for personalized therapy. By leveraging machine learning algorithms, metasurface parameters such as phase distribution, resonance frequency, or polarization response can be dynamically tuned in response to biofeedback signals like temperature, pH, or local electrical activity. This allows for precise, context-aware modulation of biological tissues, making metasurface-assisted biointerfaces responsive to patient-specific physiological changes. Figure 4 highlights key future directions in metasurface-assisted bioelectronics. Advancements include cortical implants with metasurface-enabled light delivery for precise neural stimulation (Fig. 4a), multifunctional and multiplexed metasurface platforms enable simultaneous sensing and modulation, supporting integrated, miniaturized biointerfaces for real-time, closed-loop therapeutic applications^92,93^, (Fig. 4b), and metasurface-driven photobiomodulation targeting cytochrome C pathways or inducing photothermal effects for neural and cancer therapies (Fig. 4c)^94,95^. An autonomous AI design framework (Fig. 4d) supports rapid, application-specific metasurface optimization, enabling the development of personalized and minimally invasive therapeutic technologies.

## Data Availability

There is no data to declare separately.
